# Antimicrobials from Venomous Animals: An Overview

**DOI:** 10.3390/molecules25102402

**Published:** 2020-05-21

**Authors:** Tania Yacoub, Mohamad Rima, Marc Karam, Jean-Marc Sabatier, Ziad Fajloun

**Affiliations:** 1 Department of Biology, University of Balamand, Kalhat, Al-Kurah, P.O. box 100 Tripoli, Lebanon; yacoubt@outlook.com (T.Y.); marckaram1@gmail.com (M.K.); 2 Institut de Génétique et de Biologie Moléculaire et Cellulaire (IGBMC), INSERM U964, CNRS U7104, Université de Strasbourg, 67400 Illkirch, France; mohamad.rima@hotmail.com; 3 Université Aix-Marseille, Institut de NeuroPhysiopathologie, UMR 7051, Faculté de Médecine Secteur Nord, 51, Boulevard Pierre Dramard-CS80011, 13344-Marseille CEDEX 15, France; 4 Faculty of Sciences 3, Lebanese University, Michel Slayman Tripoli Campus, Ras Maska 1352, Lebanon; 5 Laboratory of Applied Biotechnology (LBA3B), Azm Center for Research in Biotechnology and its Applications, Doctoral School for Sciences and Technology, Lebanese University, El Mittein Street, 1300 Tripoli, Lebanon

**Keywords:** snake, frog, bee, scorpion, ant, spider, antimicrobial activities

## Abstract

The inappropriate or excessive use of antimicrobial agents caused an emerging public health problem due to the resulting resistance developed by microbes. Therefore, there is an urgent need to develop effective antimicrobial strategies relying on natural agents with different mechanisms of action. Nature has been known to offer many bioactive compounds, in the form of animal venoms, algae, and plant extracts that were used for decades in traditional medicine. Animal venoms and secretions have been deeply studied for their wealth in pharmaceutically promising molecules. As such, they were reported to exhibit many biological activities of interest, such as antibacterial, antiviral, anticancer, and anti-inflammatory activities. In this review, we summarize recent findings on the antimicrobial activities of crude animal venoms/secretions, and describe the peptides that are responsible of these activities.

## 1. Introduction

The world is facing an increase in antimicrobial resistance, leading to an irreversible increase in the strains of microbes, mainly bacteria, that are resistant. This is due to the misuse and overuse of antimicrobials in humans, animals, and plants. The most commonly known antimicrobials are antibiotics, which are becoming less effective in treating diseases. In 2017, the World Health Organization (WHO) published a list about the threat of different classes of bacteria that are resistant to multiple antibiotics in an effort to encourage the development of new antibiotics [1]. However, an update posted by the WHO on 13th January 2020 shows that drug-resistant infections are still a big threat due to a lack of novel antibiotics [2]. In the US, for example, more than 2.8 million antibiotic-resistant infections occur yearly, killing over 35,000 people [3]. Antimicrobial resistance is a global crisis; in fact, in their most recent released report, the UN Interagency Coordination Group (IACG) on antimicrobial resistance (AMR) stated that drug-resistant diseases are causing at least 70,000 deaths globally. By 2050, 10 million deaths are expected per year. Multidrug-resistant organisms not only pose a significant threat to intensive care patients but also to residents of long-term care facilities; hence, the importance of the implementation of effective infection control measures [4]. Due to the severity of the situation, there has been an urgent need to develop less toxic and more effective natural agents with different mechanisms of action against these microbial infections. Nature has been known to offer many pharmacological effective compounds, and thus, is a rich source of natural antimicrobials that are being widely investigated for their potential use as alternatives to over-the-counter antimicrobials. As such, animal venoms, and algae and plant extracts have been widely explored for their content in antimicrobial compounds.

Venomous animals are found in many phyla, such as Cnidarians (sea anemones, jellyfish, and corals), Echinodermata (starfishes and sea urchins), Molusca (cone snails), Arthropods (arachnids and insects), and most importantly, Chordata (reptiles, fishes, and amphibians) [5]. Venomous animals possess a special apparatus for injecting venom as a tool of self-defense or for immobilizing preys. Many drugs have been developed from their venom; Captopril^®^, for example, is an antihypertensive drug derived from an angiotensin converting enzyme inhibitor from snake venom [6]; and Prialt^®^, from cone snails’ venom, is used to treat people who suffer from severe chronic pain [7]. Other studies focused on the development of antimicrobial peptides (AMPs) derived from animal venoms. AMPs are biologically active peptides of less than 100 amino acid residues, which are either formed by alpha-helices, beta-sheets, extended structures, or disordered loops, and can be cationic, anionic, or amphipathic [8]. They have been found in all invertebrates, including insects and marine organisms, as well as in a variety of vertebrates like fish, mammals, and amphibians. Similar to plants, invertebrates lack an adaptive immune system and depend on the innate immune system for protection; thus, these peptides serve as their primary defense mechanism. In other species, AMPs serve as adjuvants to the already established innate and adaptive immune systems. In other words, antimicrobial peptides are ancient weapons that transverse the evolutionary spectrum by allowing the successful evolution of multicellular organisms. They have proved to be highly effective against pathogens with little resistance due to their membrane-disruptive mechanisms [9]. In scorpions, for example, the venom gland in the last postabdominal segment is in direct contact with the environment, which might lead to contamination by soil organisms in the absence of these peptides [10]. AMPs are able to play a dual role; other than protecting animals from infections, they assist in the predatory process, including paralysis and digestion of their prey. Their antimicrobial properties have led to them being considered potential antibiotics [11]. AMPs have different mechanisms of action; they either lead to pore formation followed by the release of intracellular content and cell death, or cause the disruption of the cell membrane and thus the lysis of the microbial cell. The energy-independent mechanism of action of AMPs is through their release from vesicles formed by the invagination of the plasma membrane [12]. Their multiple mechanisms of action reduce resistance development by the microorganism. However, since they are isolated in a small amount, chemical synthesis processes, which also confer better potency, selectivity, and stability to these AMPs, are a must [13]. As such, thousands of AMPs have already been discovered and archived in the Antimicrobial Peptide Database [14].

**Figure 1:**
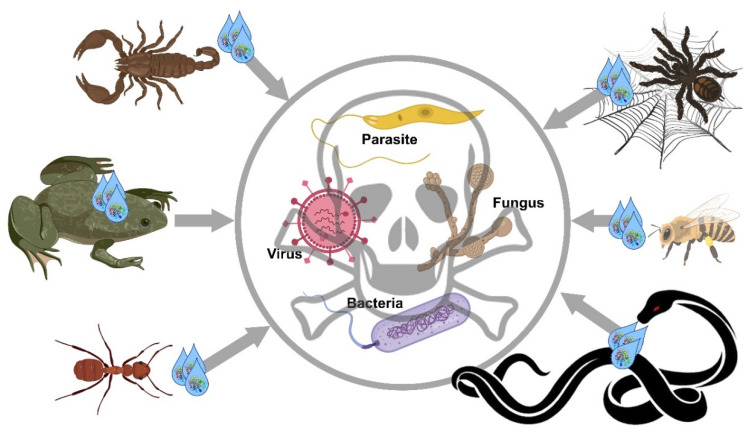
Schematic representation of the main animal venoms/secretions presented in this review for their antimicrobial properties, including antiparasitic, antiviral, antibacterial, and antifungal activities.

This review summarizes the recent findings on antimicrobial agents, mainly peptides (Table 1), but also whole venom or other components, derived from different venomous arthropods or vertebrates. These antimicrobial agents can target bacteria, fungi, parasites, or viruses (Figure 1).

## 2. Antimicrobial Activities of Animal Venoms and Secretions

### 2.1. Antimicrobial Agents from Spiders

Spiders form a wide class of arachnids with 48,409 known species so far [15]. For most people, the thought of spiders triggers extreme fear; however, the vast majority of spiders are harmless. Potentially lethal bites are limited to some taxa, such as widow spiders (Latrodectus sp., Theridiidae) and armed spiders (Phoneutria sp., Ctenidae) [16]. Spiders are known to produce silk that is known for its strength and toughness and thus has been an interesting material for scientists. In fact, many studies have shown that spider webs significantly exhibit antimicrobial activities. Phartale et al. proved that *Pardosa berivivulva* silk has useful bactericidal and fungicidal properties [17]. In agreement, antimicrobial activities were also reported for *Tegenaria domestica*’s web extract [18]. On the contrary, in a more recent study, other spider silk from *Linothele fallax* and *Linothele megatheloids* did not show any antibacterial properties [19], suggesting that antimicrobial properties are not common in all spider silks. Venom from spiders consists of salts, acylpolyamines, peptides, enzymes, amino acids, nucleotides, and low molecular mass compounds [20]. In addition to its neurotoxic effects, spider venom can exhibit antiarrhythmic, analgesic, antitumor, and most importantly, antimicrobial activities. In fact, whole venom from *Agelena labryunthica* was able to cause loss of cytoplasm, cell wall depression, and cell shrinkage of many strains of bacteria [21]. Venom components responsible for this antimicrobial activity were also investigated. This includes acylpolyamines, such as the polyamine toxin VdTX-I from *Vitalius dubius* [22], and low molecular mass molecules, such as those extracted from Laxosceles spider, which affected the virulence of *Pseudomonas aeruginosa* strains [23]. Many other antimicrobial peptides were identified from spider venom. For example, an antimicrobial peptide from the ant spider *Lachesana tarabaevi* was able to significantly decrease the viability of *Chlamydia trachomatis* [24]. The first spider AMPs, named lycotoxins-I and II from *Lycosa carolinensis*, had both antibacterial and antifungal activities by inhibiting the growth of bacteria and *Candida glabrata* yeasts. Lycotoxins, considered as pore-forming peptides, caused Ca^2+^ efflux from rat synaptosomes, induced hemolysis of erythrocytes, and dissipated voltage gradients across the muscle membrane [25]. Similarly, Oh-defensin from the venom of *Ornithoctonus hainana* displayed antimicrobial activities against Gram-positive and Gram-negative bacteria as well as against fungi [26]. Peptides from the venom of spiders are also able to display antiparasitic effects, Lycosin-I derived from the venom of the spider *Lycosa singoriensis* had antiparasitic effects against *Taxoplasma gondii* both in vitro and in vivo [27]. This peptide exhibits rapid and broad-spectrum bactericidal activity by interacting directly with the bacterial cell membrane [28]. In addition, spider venom peptides are able to exert antiviral effects. Av-LCTX-An1a is a defense peptide from the venom of the *Alopecosa nagpag* spider that presents antiviral activities against flavivirus infection by acting as an inhibitor of the viral protease NS2B-NS3 [29]. The presence of these antimicrobials has driven scientists to use them as templates for the production of novel antibiotics. To improve their efficiency, synthetic peptides derived from naturally occurring AMPs are slightly modified. As such, LyeTxI-b, a peptide derived from LyeTxI from the venom of the spider *Lycosa erythrognatha*, has improved the antibacterial activity both in vitro and in vivo. The removal of a single amino acid residue and the acetylation of the N-terminus of the peptide are responsible for its enhanced action [30]. Together, these studies prove that spiders are a rich source of antibacterial, antiviral, antiparasitic, and antifungal compounds.

### 2.2. Antimicrobial Agents from Scorpions

Like spiders, scorpions are also arthropods belonging to the class of Arachnids. These venomous animals are present everywhere except Antarctica. Although we rarely think of scorpions as being beneficial because of the fatality of their stings, they have been used in traditional medicine. For more than 2000 years, *Buthus martensii Karsch* has been used as a traditional Chinese medicine because of its outstanding pharmaceutical effects [31]. Androctonin, a peptide isolated from the hemolymph of scorpions of the species *Androctonus australis*, proved active against both bacteria and fungi [32]. The peptide, following an electrostatic interaction with the target membrane, loses its β-sheet organization and lies parallel to the lipid monolayer, causing its permeabilization and subsequent potassium ions efflux [33]. Therefore, scorpions’ organs or venoms have been found to be effective in treating conditions, such as cancer [34]. Scorpion venoms are mixtures of peptides, enzymes, mucoproteins, free amino acids, amines, nucleotides, lipids, heterocyclic components, inorganic salts, and other unknown substances. Many antimicrobial peptides from the venom of scorpions have been identified. These AMPs are cationic, amphipathic, and alpha-helical peptides able to target bacteria and fungi by causing membrane lysis. The first discovered AMP was a scorpion defensin from *Leiurus quinquestriatus* [35]. Since then, many other peptides with antibacterial activities have been discovered. Hadrurin from the venom of the scorpion *Hadrurus aztecus* demonstrated antibacterial activity against many strains of bacteria through a membrane destabilization mechanism [36]. Heteroscopine-1 (HS-1) is another peptide derived from the venom of the scorpion *Heterometrus laoticus* that is effective in eradicating *Bacillus subtilis*, *Klebsiella pneumonia*, and *Pseudomonas aeruginosa* [37]. Most peptides from scorpion venom are able to display both an antibacterial and antifungal/or antiparasitic effect. This includes stigmurin, an AMP from the venom gland of *Titys stigmurus* that disrupt target membranes [38], and pandinin 1 and pandinin 2 from the venom of the scorpion *Pandinus imperator*. The latter two presented high antimicrobial activities, via their pore-forming activity, against a range of Gram-positive bacteria, with only pandanin 2 affecting *Candida albicans* [39]. Scorpine, another peptide isolated from the scorpion *Pandinus imperator*, has high sequence homology to HS-1 and shows antibacterial and antimalarial effects [40]. Other than the antibacterial, antifungal, and antiparasitic activities, peptides from scorpion venom are also able to affect viruses. Smp76 is a peptide purified from the venom of *Scorpio maurus palmatus* that has antiviral activity, through the interaction with viral particles of hepatitis C virus (HCV), dengue virus (DENV), and viruses belonging to the Flaviviridae family [41]. As discussed previously, the potential clinical use of AMPs can be optimized by improving their activity in synthetic peptides derived from AMPs. Mucroporin-M1 is an engineered peptide designed from the sequence of mucroporin from *Lychas mucronatus*. It was synthesized by replacing the amino acid residues, at the hydrophilic side of the alpha-helix, with positively charged residues. As compared to the original peptide, mucroporin-M1 showed improved antibacterial activity against Gram-negative and Gram-positive bacteria, and most importantly, against antibiotic-resistant strains, such as methicillin-resistant *Staphylococcus aureus* [42]. Additionally, biosynthetic precursors of two peptides AaeAP1 and AaeAP2 from the venom of *Androctonus aeneas* displayed broad-spectrum antimicrobial properties. The increase in the net positive charge of these analogues facilitates their interaction with the target cell membranes [43]. Two analogs of the peptide stigmurin from the *Tityus stigmurus* venom gland named StigA6 and StigA16 revealed increased antimicrobial activity and less toxicity on the normal cell line, while retaining the activity of the native peptide on cancer cells [44].

### 2.3. Antimicrobial Agents from Wasps and Bees

Wasps and bees are closely related flying insects that belong to the order *Hymenoptera* and the suborder *Apocrita.* Their venoms are also composed of proteins, enzymes, peptides, and other smaller molecules. The common proteins between wasp and bee venoms include phospholipases A2 and B, hyaluronidases, and phosphatases; however, melittin, apamin, and mast cell degranulating peptides are exclusive to bees, and mastoparan and bradykinin peptides are exclusive to wasps [45]. As any other envenomation, bee and wasp stings can employ toxic effects. However, researchers have shown that bee and wasp venoms have many pharmacological properties. In fact, bee venom has been used as a traditional medicine to treat conditions, such as cancer and Parkinson’s disease [46]. Bee venom-derived products have already been commercialized. For example, Apitox is an FDA-approved (FDA, for Food and Drug Administration) product for the relief of pain and swelling associated with inflammatory diseases, such as rheumatoid arthritis and multiple sclerosis. It consists of purified bee venom from *Apis mellifera* [47]. Whole venom from bees and wasps has been proven to exhibit antimicrobial activities. For instance, the wasp’s *Vespa orientalis* crude venom efficiently inhibited the growth of Gram-positive and Gram-negative bacteria with a higher affinity against Gram-positive bacteria. In fact, wasp venom peptides possess positively charged amphipathic secondary structures that are able to interact with the anionic components of the bacterial membrane. It is the difference in the cell envelope structure that explains the predominant effect of the venom on Gram-positive bacteria [48]. In another study, the venom of the same wasp showed antifungal activity against the fungal strain *Candida albicans* [49]. Whole venom of the honey bee *Apis mellifera* exhibited antibacterial and anti-inflammatory activities against the skin bacteria *Propionibacterium acnes*, and thus can be potentially used to treat acne vulgaris [50]. It is also important to mention that other bee components have potent antimicrobial activities. Besides its taste and nutritional value, honey has antibacterial [51] and antiviral effects [52]. Peptides responsible for these antimicrobial activities have been isolated from wasp and bee venoms. In one study, nine AMPs were identified from the venom gland of the wasp *Vespa tropica* and were classified into two different families: Mastoparan and vespid chemotactic peptides (VCPs). They were able to exert broad-spectrum activity against standard and clinically isolated strains of bacteria [53]. Two antibacterial peptides named dominulin-A and dominulin-B were also found in the venom and on the surface of the female cuticle of the social wasp *Polistes dominulus* [54]. Another peptide, melittin, was purified from *Apis mellifera* bee venom and has been extensively reviewed for its antibacterial activity. Melittin is composed of 26 amino acid residues with amphipathic characteristics, allowing it to interact with lipid membranes and increase their permeability. The formation of pores along with the leakage of atomic ions and molecules causes cell lysis [55]. Melittin shows very promising minimum inhibitory concentration (MIC) values from 4 to 40 µg/mL against several strains of bacteria [56]. In other studies, the antibacterial activity of melittin was reported against methicillin-resistant *Staphylococcus aureus*-infected mice [57] and *Borrelia burgdoferi* [58]. Emerging evidence suggests that melittin’s antimicrobial activity displays mechanisms of action that do not rely on the peptide’s ability to damage the microbes’ cell membranes. This was the case of the inhibition of methicillin-resistant *Staphylococcus aureus* infections by melittin, which was able to potentiate anti-inflammatory responses and wound healing [57]. Regarding the antifungal and antiparasitic peptides from bees and wasps, mastoparan from the venom of the wasp *Polybia paulista* does not only show antibacterial effects but can also inhibit the developmental forms of the parasite *Trypanosoma cruzi* by inhibiting its vital enzyme glyceraldehyde-3-phosphate dehydrogenase [59]. Another mastoparan peptide from the venom of *Pseudopolybia vespiceps* presented significant antifungal activities against *Candida albicans* and *Candida neoformans* [60]. Bee venom melittin is also able to induce antiparasitic activities. In fact, melittin from *Apis mellifera* acted in vitro against promastigotes and intracellular amastigotes of *Leishmania infantum*. The results suggested that melittin can indirectly eliminate the parasite through a macrophage immunomodulatory effect [61]. Bee venom is also known to exhibit antifungal activities. Interestingly, this activity proved to be stronger than that of fluconazole, a commercial antifungal drug [62]. Park et al. showed that only bee venom in the whole form and not in its separated form is effective against the fungus *Trichophyton rubrum* [63], suggesting a synergy between venom compounds. Antimicrobial peptides from wasps and bees also include peptides with antiviral activities. A mastoporan-derived peptide MP7-NH2 was able to inactivate a range of enveloped viruses in vitro by inserting itself into the viral lipid envelope and causing its disruption [64]. Bee venom from *Apis mellifera* and its component melittin inhibited the replication of some enveloped and non-enveloped viruses in vitro and melittin protected mice challenged with lethal doses of influenza A virus H1N1 [65]. Finally, some of these antimicrobial peptides are either being optimized or are currently used in biotechnological productions. This is the case of melittin that is used in the coating of medical devices, such as contact lenses, to prevent the growth of undesirable microorganisms [45].

### 2.4. Antimicrobial Agents from Ants

Together with wasps and bees, ants belong to the class of Hymenoptera but are part of the medically important group Formicidae [66]. They are an abundant group of venomous organisms with more than 13,000 species that dominate most environments. Stinging ant venoms are composed of many toxins and other types of molecules, including proteins, peptides, hydrocarbons, alkaloids, free amino acids, biogenic amines, formic acid, salts, and sugars [67], with the exception of the stinging ants of the genera *Solenopsis* and *Monomorium* that produce a venom with few proteins and a high alkaloid content [68]. Despite the small size of ants resulting in small quantities of venom, which limits studies, ant venom was reported to have many therapeutic uses. In fact, *Polyrhachis lamellidens* presented remarkable analgesic and anti-inflammatory activities, which supported its use as a Chinese medicinal ant against inflammatory diseases [69]. Ant venom also displayed an antitumor effect on human breast carcinoma cells [70]. Among these biological activities of interest, peptides from ant venom also possess an antimicrobial effect. Several peptides named ponericins were identified from the venom of the ant *Neoponera goeldii* (formerly known as *Pachycondyla goeldii*) and were classified into three families: Ponericins G, W, and L. These peptides have defensive roles against microbial pathogens, such as Gram-positive and Gram-negative bacteria, and they act by targeting the cell membrane. Similarities between ponericins and other peptides provided insights into their mechanisms of action. While ponericins W share a 70% sequence similarity with melitin that acts via the formation of transmembrane pores, ponericins G and L share similarities with cecropins and dermaseptins that use a “carpet-like” mechanism for membrane disruption [71]. Three linear antibacterial peptides sharing a low similarity to ponericin peptides were also isolated from the venom of the ant *Ectatomma quadridens* [72]. Peptides derived from ant venom have also shown antifungal activities. For example, pilosulin-1, a cytotoxic peptide from the venom of the ant *Myrmecia pilosula*, does not only display potent antibacterial activity against standard and multidrug-resistant Gram-positive and Gram-negative bacteria, but IT is also active against *Candida albicans* [73]. The cytotoxic and antimicrobial activities of pilosulin-1 are due to its net cationicity, which allows its interaction with the negatively charged microbial surface, as well as its ability to assume an amphipathic alpha-helical conformation, which facilitates the incorporation of the peptide into the target membrane, causing its lysis [74]. Moreover, sting secretions from *Crematogaster scutellaris*, which are a mixture of compounds produced by the venom glands and the Dufour’s gland, were able to strongly inhibit the growth of bacteria and entomopathogenic fungi [75]. Additionally, Benmoussa et al. showed that P17, a host defense peptide from the ant venom of *Tetramorium bicarinatum*, promoted the antifungal activities of macrophages by triggering the production of reactive oxygen species (ROS) and the release of inflammosome-depedent interleukin (IL)-1β, which are critical for this fungicidal activity [76]. Within the same ant’s venom, bicarinalin was characterized as the most abundant peptide in the venom. Bicarinalin, through a membrane permeabilization mechanism, showed broad-spectrum antibacterial and antifungal activities as well as an antiparasitic effect against *Leishmania infantum* [77].

### 2.5. Antimicrobial Agents from Snakes

Snakes are reptiles belonging to the suborder Serpentes found on every continent except Antarctica, with around 3789 species to date [78]. The most complex animal venoms are those of snakes, with the most studied families being Vipiridae, Crotalidae, and Elapidae. Snake venom contains mostly enzymes and non-enzymatic proteins and/or peptides among other components, including lipids, metals, and ions. Although being venomous, snakes have always been associated with healing. The Rod of Asclepius, which is the authentic symbol of medicine, represents a staff with a sacred snake coiled around it [79]. Despite the undesirable effects of snake envenomation due to the hemotoxic, neurotoxic, and cytotoxic nature of the venom, snake venom components have been well studied for their desirable pharmacological effects [80]. Many hypotheses have been tested and validated, leading to beneficial discoveries in the pharmaceutical field. Besides Captopril^®^, many other drugs have been developed from snake venom, including the FDA-approved antiplatelet drugs Tirofiban and Eptifibatide [81]. Regarding the activities of interest of this review, different snake components have also been studied for their antimicrobial potential. This comes from the fact that snake bite victims do not suffer from wound infections. Crotamine, a small basic myotoxin from *Crotalus durissus*, is an AMP that targets bacteria through membrane permeabilization [82]. The enzymes phospholipase A2 (PLA2) and L-amino-acid oxidase (L-AAO) are also responsible for conferring the venom its antimicrobial effects. L-AAO from *Bothrops marajoensis* venom inhibited the growth of *Pseudomonas aeruginosa*, *Candida albicans*, and *Staphylococcus aureus*. Additionally, L-AAO as well as whole venom inhibited the parasitic growth of *Leishmania chagasi* and *Leishmania amazonensis* [83]. PLA2 and L-AAO from the Lebanese viper, *Montivipera bornmuelleri*, as well as its crude venom, exhibited strong antibacterial activities [84,85,86]. L-AAOs have been suggested to act via the generation of hydrogen peroxide through the oxidative action of these enzymes, thus causing oxidative stress in the target cell [87]. PLA2s exert their bactericidal effects through membrane permeabilization and a subsequent damage mechanism [88].

Cathelicidins are the main family of naturally occurring antimicrobial peptides from snake venom, which were first discovered by Zhao et al., and have been widely studied for their antibacterial activity through the membrane disruption of microorganisms [89]. Their activities were reported against multidrug-resistant *Acinetobacter baumannii* and methicillin-resistant *Staphylococcus aureus*, and was more efficient than that of nine routinely used antibiotics [89]. Cathelicidin from the venom of *Bungarus fasciatus* also exerted antibacterial activity [90]. Another peptide isolated from the venom of *Naja atra* proved active against clinical isolates of multidrug-resistant strains of *Mycobacterium tuberculosis* [91]. Recombinant omwaprin, a 50-amino acid cationic peptide from the venom of *Oxyuranus microlepidotus*, showed selective species-specific and dose-dependent antibacterial activity against Gram-positive bacteria via a membrane disruption mechanism [92]. Crotamine is a mycotoxin that has structural similarities with the AMPs β-defensins, which suggests that it can also function as an AMP. Yamane et al. validated this hypothesis by showing that at low concentrations, crotamine from *Crotalus durissus terrificus* is a potentially valuable candicidal agent [93]. It has a positive net charge on its surface, allowing it to bind to negatively charged membranes, leading to the perturbation of a significant portion of the target membrane, disruption, and subsequent rupture. This mechanism does not involve membrane permeabilization [94]. Pep5Bj, a peptide isolated from *Bothrops jararaca* venom, showed high inhibitory effects against *Fusarium oxysporum*, *Colletotrichum lindemuthianum*, and *Saccharomyces cerevisiae*. The antibacterial activity of Pep5Bj was proved to be related to membrane permeabilization [95]. The discussed peptides from snake venom not only present antibacterial and antifungal effects, but they also exhibit antiparasitic activity. A cathelicidin-related peptide from *Crotalus durissus terrificus* venom, called crotalicidin, was able to inhibit all *Trypanosoma cruzi* developmental forms through necrosis, as determined by the loss of membrane integrity and cell shrinkage [96]. Additionally, crotamine from the South American rattlesnake (*Crotalus durissus terrificus*) presented potent antiplasmodial activity in culture. Interestingly, crotamine was able to internalize into *Plasmodium falsiparum*-infected but not intact erythrocytes. This may be due to the preferential affinity between the acidic peptide and infected erythrocytes, which have increased negative charge due to the host cell remodeling mediated by parasites. The peptide was detected in the parasite nucleus and parasitophorous vacuole, suggesting the disruption of parasite acidic compartments’ H^+^ homeostasis in the crotamine antimalarial mechanism of action [97]. Snake venom L-AAO, PLA2, and metalloproteases have all shown antiviral activities against human immunodeficiency virus (HIV) and Dengue virus (DENV) [98]. For example, PLA2s from the venom of *Bothrops leucurus* decreased the amounts of DENV viral RNA in infected cells [99]. Similar to AMP from other animals, snake venom-derived AMPs were used as templates to synthetize optimized versions of these peptides with improved functions. For example, two AMPs (omw 1 and omw 2), designed based on the snake venom peptide omwaprin, proved to be less toxic and potent antimicrobial agents, and thus can be considered for further studies [100].

### 2.6. Antimicrobial Agents from Frogs and Toads

Frogs and toads are amphibians belonging to the order Anura and can be distinguished by their appearance. Most frogs have long legs and smooth mucus-covered skins, while toads have shorter legs and thicker skins. Toads mainly belong to the family Bufonidae. Compounds found in their glands are biogenic amines, bufodienolides, alkaloids, steroids, peptides, and proteins. Toad skin secretions have been used in traditional Chinese medicine for hundreds of years. Dried secretions from the auricular and skin glands of the Chinese toad *Bufo bufo gargarizans* (toad venom) were used to treat inflammation and infections. Additionally, huachansu, which is the sterilized hot water extraction of the dried toad skin, was successfully used to treat several types of cancer [101]. Even fresh toad venom from *Bufo gargarizans* was found to contain active antitumor components [102]. Similarly, frog venom has many therapeutically beneficial applications. For example, Chang et al. showed that a peptide homolog of prokineticins from the venom of the frog *Amolops Jingdongenesis* is a potent wound healing regulator [103].

Amphibian skin is well known for exerting functions of particular importance, such as respiration, water regulation, excretion, and temperature control. This means that the skin of frogs and toads is also directly exposed to the environment and thus to microorganisms, which explains its use as a defense strategy. For instance, major components from the skin secretion of the toad *Bufo rubescens* revealed antimicrobial activity. Skin AMPs are able to exert their function by permeating and destroying the plasma membrane of microorganisms [104]. The steroids telecinobufagin and hellebrignin, from the parotid macrogland secretions of the toad *Rhinella jimi*, displayed potent antiparasitic activity against *Leishmania chagasi* promastigotes through the induction of mitochondrial damage and plasma membrane disturbance, leading to cellular death [105]. The skin secretions of many anurans also contain peptides with antimicrobial activity. This mechanism arose early in the evolution of these anurans. Eight peptides named ascaphins, with broad-spectrum antibacterial activity, were isolated from the skin secretions of the most primitive extant frog *Ascaphus truei* [106]. Syphaxin (SPX) is another antibacterial peptide that was isolated from the skin secretions of the frog *Leptodactylus syphax* [107]. More recently, a novel peptide named *Limnonectes fujianensis* Brevinvin (LFB) from the skin secretion of the frog *Limnonectes fujianensis* displayed potent antibacterial and antifungal activities by its ability to insert itself into the lipid bilayer [108]. Maximins are examples of antimicrobial peptides found on the skin of the toad *Bombina maxima* [109]. Megin 1 and megin 2, purified from the skin venom of the spadefoot toad *Megophrys minor*, showed antimicrobial capacities against bacteria and fungi [110]. Together, these findings highlight the wealth of frogs’ and toads’ skin secretions in bioactive compounds with antimicrobial interest. Analogs of these peptides were developed to reduce toxicity against human erythrocytes, hepatoma-derived cells, and fibroblasts, while retaining their antibacterial and antifungal activities [111]. Temporins are short hydrophobic peptides synthesized in the skin of various frogs of the Ranidae family and known to possess antibacterial and antifungal activities due to their ability to perturb the integrity of the target cell membrane [112]. An analogue of temporin, a defense peptide from the frog *Rana temporaria*, was constructed by a one-point mutation in the temporin sequence and two extra lysines at the N-terminal. This modification abolished the hemolytic activity of the peptide without affecting its activity against Gram-positive and Gram-negative bacteria [113].

## 3. Conclusions

Antimicrobial resistance is triggered by inappropriate or excessive prescription of antibiotics or through overuse in agriculture. The resulting resistant microbes are clearly an emerging public health problem. As antimicrobial resistance is spreading throughout the world, the discovery of new substances is mandatory to fight against it. Naturally occurring medicinal products have been used for ages. In fact, the vast diversity of bioactive molecules in nature has long inspired scientists in their search for potential therapeutic agents. More recently, there has been a resurge in the use of AMPs due to the decrease in the efficiency of common treatments. AMPs are able to target a broad spectrum of microbes with little resistance and can have a synergetic effect with antibiotics. As previously mentioned, components of venom have therapeutic benefits, justifying their use in traditional medicine. Animal venom is thus a particularly promising source in this search for new antimicrobial compounds. Many AMPs from venom have shown high efficacy in vitro and in vivo, but challenges to overcome their host toxicity, hemolytic activity, as well as the bioavailability and stability of these peptides are still present. This results in the need for profitable production and physiochemical optimization of these AMPs when needed. Many studies are now investigating synthesized analogues of AMPs. They rely on the advantages of bioinformatics and in silico tools that help in the prediction of peptide-host interactions to find the peptide sequence with the least possible secondary effects before launching its synthesis. With more than 100,000 venomous animals, naturally occurring antimicrobial agents present in venomous species thus hold promises for the development of novel therapeutic agents. Currently, only few antimicrobial peptides are present on the market for tropical use, such as Gramicidins and Polmyxin B; however, the development of new drugs out of AMP analogs continues with different strategies: (i) Prodrug and peptide conjugate construction, (ii) combination of AMPs with existing antimicrobials, (iii) induction of AMP expression in host cells, and (iv) AMP production in probiotic bacteria [114].

## Figures and Tables

**Table 1:** Primary sequences of antimicrobial peptides from animal venoms/secretions with their respective antimicrobial activity. The accession number/reference of each peptide is given.

Animal	Peptide	Primary Sequence	Activity	Accession Number/Reference
**Spiders**				
*Lycosa carolinensis*	Lycotoxins-I	_N_IWLTALKFLGKHAAHLAKQQLSKL_C_	Antibacterial Antifungal	P61507.1
	Lycotoxins-II	_N_KIKWFKTMKSIAKFIAKEQMKKHLGGE_C_		P61508.1
*Ornithoctonus hainana*	Oh-defensin	_N_MLCKLSMFGAVLGVPACAIDCLPMGKTGGSCEGGVCGCRKLTFKILWDKKFG_C_		[26]
*Alopecosa nagpag*	Av-LCTX-An1a	_N_METAHVFLLSFLLLCVFAVDLIEAGFGCPLDQMQCHNHCQSVRYRGGYCTNFLKMTCKCYG_C_	Antiviral	QGD15041
*Lycosa erythrognatha*	LyeTxI	_N_XIWLTALKFLGKNLGKLAKQQLAKLX_C_	Antibacterial	6CL3_A
**Scorpions**				
*Androctonus australis*	Androctonin	_N_RSVCRQIKICRRRGGCYYKCTNRPY_C_	Antibacterial Antifungal	P56684.1
*Leiurus quinquestriatus*	Defensin	_N_GFGCPLNQGACHRHCRSIRRRGGYCAGFFKQTCTCYRN_C_	Antibacterial	P41965.1
*Hadrurus aztecus*	Hadrurin	_N_GILDTIKSIASKVWNSKTVQDLKRKGINWVANKLGVSPQAA_C_		P82656.1
*Pandinus imperator*	Pandinin 1	_N_GKVWDWIKSAAKKIWSSEPVSQLKGQVLNAAKNYVAEKIGATPT_C_		P83239.1
	Pandinin 2	_N_FWGALAKGALKLIPSLFSSFSKKD_C_	Antibacterial Antifungal	P83240.1
	Scorpine	_N_MNSKLTALIFLGLIAIAYCGWINEEKIQKKIDERMGNTVLGGMAKAIVHKMAKNEFQCMANMDMLGNCEKHCQTSGEKGYCHGTKCKCGTPLSY_C_	Antimalarial Antibacterial	P56972.1
*Scorpio maurus palmatus*	Smp76	_N_GWINEKKMQQKIDEKIGKNIIGGMAKAVIHKMAKNEFQCVANVDTLGNCKKHCAKTTGEKGYCHGTKCKCGIELSY_C_	Antiviral	[41]
*Lychas mucronatus*	Mucroporin	_N_MKVKFLLAVFLIVLVVTDHCHALFGLIPSLIGGLVSAFKGRRKRQMEARFEPQNRNYRKRELDLEKLFANMPDY_C_	Antibacterial	ACF93401.1
**Wasps and Bees**				
*Polistes dominulus*	Dominulin-A	_N_INWKKIAEVGGKILSSL_C_	Antibacterial	P0C1M6.1
	Dominulin-B	_N_INWKKIAEIGKQVLSAL_C_		P0C1M7.1
*Apis mellifera*	Melittin	_N_MKFLVNVALVFMVVYISYIYAAPEPEPAPEPEAEADAEADPEAGIGAVLKVLTTGLPALISWIKRKRQQG_C_	Antibacterial Antiparasitic Antiviral	AFI40556.1
*Polybia paulista*	Mastoparan	_N_IDWKKLLDAAKQIL_C_	Antibacterial Antiparasitic	P0C1Q4.1
Synthetic peptide	Mastoporan-derived peptide (MP7-NH2)	_N_INLKALAALAKALL-NH2_C_	Antiviral	[64]
**Ants**				
*Pachycondyla goeldii*	Ponericin-L1	_N_LLKELWTKMKGAGKAVLGKIKGLL_C_	Antibacterial	P82421.1
	Ponericin-L2	_N_LLKELWTKIKGAGKAVLGKIKGLL_C_		P82422.1
	Ponericin-G1	_N_GWKDWAKKAGGWLKKKGPGMAKAALKAAMQ_C_	Antibacterial Antifungal	P82414.1
	Ponericin-G2	_N_GWKDWLKKGKEWLKAKGPGIVKAALQAATQ_C_		P82415.1
	Ponericin-G3	_N_GWKDWLNKGKEWLKKKGPGIMKAALKAATQ_C_		P82416.1
	Ponericin-G4	_N_DFKDWMKTAGEWLKKKGPGILKAAMAAAT_C_		P82417.1
	Ponericin-G5	_N_GLKDWVKIAGGWLKKKGPGILKAAMAAATQ_C_		P82418.1
	Ponericin-G6	_N_GLVDVLGKVGGLIKKLLP_C_		P82419.1
	Ponericin-G7	_N_GLVDVLGKVGGLIKKLLPG_C_		P82420.1
	Ponericin-W1	_N_WLGSALKIGAKLLPSVVGLFKKKKQ_C_		P82423.1
	Ponericin-W2	_N_WLGSALKIGAKLLPSVVGLFQKKKK_C_		P82424.1
	Ponericin-W3	_N_GIWGTLAKIGIKAVPRVISMLKKKKQ_C_		P82425.1
	Ponericin-W4	_N_GIWGTALKWGVKLLPKLVGMAQTKKQ_C_		P82426.1
	Ponericin-W5	_N_FWGALIKGAAKLIPSVVGLFKKKQ_C_		P82427.1
	Ponericin-W6	_N_FIGTALGIASAIPAIVKLFK_C_	Antibacterial	P82428.1
*Myrmecia pilosula*	Pilosulin-1	_N_MKLSCLLLTLTIIFVLTIVHAPNVEAKDLADPESEAVGFADAFGEADAVGEADPNAGLGSVFGRLARILGRVIPKVAKKLGPKVAKVLPKVMKEAIPMAVEMAKSQEEQQPQ_C_	Antibacterial Antifungal	Q07932.1
*Tetramorium bicarinatum*	P17	_N_MKLSFLSLALATIFVMAIIYAPQMEARASSDADADAAASADADADALAEASALFKEILEKIKAKLGKK_C_	Antifungal	AIO11144.1
	Bicarinalin	_N_MKLSFLSLVLAIILVMALMYTPHAEAKAWADADADATAAADADADAVADALADAVAKIKIPWGKVKDFLVGGMKAVGKK_C_	Antibacterial Antifungal Antiparasitic	W8GNV3.1
**Snakes**				
*Crotalus durissus*	Crotamine	_N_MKILYLLFAFLFLAFLSEPGNAYKQCHKKGGHCFPKEKICLPPSSDFGKMDCRWRWKCCKKGSGK_C_	Antibacterial	AAF34911.1
*Bothrops marajoensis*	L-AAO	_N_AHDGNPLEECFREDDEEFFLEIAKNGLTATSNPKRVVIV_C_	Antibacterial Antifungal Antiparasitic	P0CJ40.1
*Bungarus fasciatus*	Cathelicidin	_N_MEGFFWKTLLVVGALAIAGTSSLPHKPLIYEEAVDLAVSIYNSKSGEDSLYRLLEAVSPPKWDPLSESNQELNFTMKETVCLVAEERSLEECDFQEDGVVMGCTGYYFFGESPPVVVLTCKPVGEEGEQKQEEGNEEEKEVEEEEQEEDEKDQPRRVKRFKKFFRKLKKSVKKRAKEFFKKPRVIGVSIPF_C_	Antibacterial	ACI22652.1
*Oxyuranus microlepidotus*	Omwaprin	_N_KDRPKKPGLCPPRPQKPCVKECKNDDSCPGQQKCCNYGCKDECRDPIFVG_C_		P83952.1
*Crotalus durissus terrificus*	Crotamine	_N_MKILYLLFAFLFLAFLSEPGNAYKQCHKKGGHCFPKEKICLPPSSDFGKMDCRWRWKCCKKGSGK_C_	Antifungal Antiparasitic	AAF34911.1
	Crotalicidin	_N_MQGFFWKTWLVLAVCGTPASLAHRPLSYGEALELAVSVYNGKAGEASLYRLLEAVPQPEWDPSSEGSQQLNFTLKETACQVEEERSLEECGFQEDGVVLECTGYYFFGETPPVVVLSCVPVGGVEEEEEEEEEEQKAEAENDEEVEKEKGDEEKDQPKRVKRFKKFFKKVKKSVKKRLKKIFKKPMVIGVTIPF_C_	Antiparasitic	U5KJM4.1
**Frogs and Toads**				
*Ascaphus truei*	Ascaphin-1	_N_GFRDVLKGAAKAFVKTVAGHIAN_C_	Antibacterial	P0CJ25.1
	Ascaphin-2	_N_GFRDVLKGAAKQFVKTVAGHIANI_C_		P0CJ26.1
	Ascaphin-3	_N_GFRDVLKGAAKAFVKTVAGHIANI_C_		P0CJ27.1
	Ascaphin-4	_N_GFKDWIKGAAKKLIKTVAANIANQ_C_		P0CJ28.1
	Ascaphin-5	_N_GIKDWIKGAAKKLIKTVASHIANQ_C_		P0CJ29.1
	Ascaphin-6	_N_GFKDWIKGAAKKLIKTVASSIANE_C_		P0CJ30.1
	Ascaphin-7	_N_GFKDWIKGAAKKLIKTVASAIANQ_C_		P0CJ31.1
	Ascaphin-8	_N_GFKDLLKGAAKALVKTVLF_C_		P0CJ32.1
*Leptodactylus syphax*	Syphaxin	_N_GVLDILKGAAKDLAGHVATKVINKI_C_		P85279.1
*Limnonectes fujianensis*	LFB	_N_GLFSVVKGVLKGVGKNVSGSLLDQLKCKISGGC_C_	Antibacterial Antifungal	[108]
*Bombina maxima^1^*	Maximin-1	_N_MNFKYIVAVSFLLASAYARSEENDEQSLSQRDVLEEESLREIRGIGTKILGGVKTALKGALKELASTYANGKRTAEEHEVMKRLEAVMRDLDSLDYPEEAAERETRSFNQEEIANLFTKKEKRILGPVISTIGGVLGGLLKNLG_C_		P83080.1
	Maximin-2	_N_MNFKYIVAVSFLIASAYARSEENDEQSLSQRDVLEEESLREIRGIGTKILGGVKTALKGALKELASTYVNGKRTAEDHEVMKRLEAVMRDLDSLDYPEEAAERETRGFNQEEIANLFTKKEKRILGPVISTIGGVLGGLLKNLG_C_		P83081.1
	Maximin-3	_N_MNFKYIVAVSFLIASAYARSVQNDEQSLSQRDVLEEESLREIRGIGGKILSGLKTALKGAAKELASTYLHRRRTAEEHEVMKRLEAVMRDLDSLDYPEEASERETRGFNQDEIANLFTKKEKRILGPVLSMVGSALGGLIKKIG_C_		P83082.1
	Maximin-4	_N_MNFKYIIAVSFFIASAYARSEEKDVQSLSQRDVLEEESLREIRGIGGVLLSAGKAALKGLAKVLAEKYANGKRTAEDHEVMKRLEAVMRDLDSLDHPEEASERETRGFNQEEIANLFTKKEKRILGPVLGLVGNALGGLIKKIG_C_		P83083.1
	Maximin-5	_N_MNFKYIVAVSFLIASAYARSVQNDEQSLSQRDVLEEESLREIRSIGAKILGGVKTFFKGALKELASTYLQRKRTAEEQHEVMKRLEAVMRDLDSLDHPEEASEREIRGFNQEEIANLFTKKEKRILGPVISKIGGVLGGLLKNLG_C_		P83084.1
*Megophrys minor*	Megin 1	_N_FLKGCWTKWYSLKPKCPF-NH2_C_		[110]
	Megin 2	_N_FFVLKFLLKWAGKVGLEHLACKFKNWC_C_		[110]
*Rana temporaria*	Temporin-A	_N_FLPLIGRVLSGIL_C_	Antibacterial	P56917.2
	Temporin-B	_N_LLPIVGNLLKSLL_C_		6GIL_A
	Temporin-C	_N_LLPILGNLLNGLL_C_		P56918.2
	Temporin-E	_N_VLPIIGNLLNSLL_C_		P56920.2
	Temporin-F	_N_FLPLIGKVLSGIL_C_		P56921.2
	Temporin-K	_N_LLPNLLKSLL_C_		P56923.2
	Temporin-L	_N_FVQWFSKFLGRIL_C_		P57104.1

Only some examples of maximins are cited in this table; however, more than 40 peptides have been identified and are accessible in the NCBI protein database.
